# Immune Consequences of Chlamydia Infections in Pregnancy and In Vitro Fertilization Outcome

**DOI:** 10.1155/S1064744996000312

**Published:** 1996

**Authors:** M. Askienazy-Elbhar

**Affiliations:** Laboratoire de Biologie Médicale Magenta 41 Boulevard de Magenta Paris 75010 France

## Abstract

This review addresses the immune consequences of Chlamydial infections in pregnancy and in vitro fertilization (IVF) outcome. In pregnancy, many works have shown the risk for perterm labor, preterm birth, or miscarriage with current infection, and stress the need for screening and treatment early in pregnancy. IVF outcome needs to be studied in relation to specific markers like inflammatory cytokines and secretory antibodies to the 60 kD heat shock protein (hsp60) and to Chlamydia, to determine their ability to predict and influence pregnancy outcome.


Infectious Diseases in Obstetrics and Gynecology 4:143–148 (1996)
© 1996 Wiley-Liss, Inc.

### Immune Consequences of Chlamydia Infections in Pregnancy and In Vitro Fertilization Outcome

**M. Askienazy-Elbhar**

*Laboratoire de Biologie Médicale Magenta, 41 Boulevard de Magenta, 75010 Paris, France*

ABSTRACT

**This review addresses the immune consequences of Chlamydial infections in pregnancy and in vitro fertilization (IVF) outcome. In pregnancy, many works have shown the risk for perterm labor, preterm birth, or miscarriage with current infection, and stress the need for screening and treatment early in pregnancy. IVF outcome needs to be studied in relation to specific markers like inflammatory cytokines and secretory antibodies to the 60 kD heat shock protein (hsp60) and to Chlamydia, to determine their ability to predict and influence pregnancy outcome.** © 1996 Wiley-Liss, Inc.

KEY WORDS
preterm birth, ectopic pregnancy, spontaneous abortion, hsp60 antibodies, inflammatory cytokines, embryo implanation.

Chlamydial infection elicits an immune response involving humoral and cellular immunity. Mild or recent infection can be controlled by neutralizing antibodies like IgG and IgA including secretory IgA.

Untreated or unsuspected infections can become chronic, and cause damage related to inflammation through cellular immune activation. Inflammatory cytokines are released as a consequence of prolonged antigen presentation and may not be sufficient to resolve long ongoing infections but are sufficient enough to maintain an inflammatory state. In pregnancy and IVF outcome, sequelae differ according to the specific immune responses that are induced.

### PREGNANCY

Chlamydial cervicitis of recent origin, in the absence of tubal damage, does not impair fertilization or egg implantation. Detection prevalence in pregnancy varies from 6%–40% according to the studies, settings and populations. Seroprevalence varies from 15%–35% according to the populations. The risk for preterm labor and preterm birth[1,2,3] but not premature rupture of membranes[1,4] is significantly higher in pregnant women with a positive chlamydial detection and increases further when bacterial vaginosis is also present. Intra-amniotic chlamydial infection can persist with intact membranes and without clinical symptoms. Antichlamydial antibodies have also been associated with perinatal complications.[5–7] If exposure occurs during pregnancy, it may be permitted by immunosuppression and/or cervical ectopy. Chorioamnionitis can occur with cytokine emission in the amniotic fluid, promoting prostaglandin PGE2 secretion and ocytocic sequelae. Hsp60 expression have been found identical in normal pregnancies and preterm birth by immunocytochemistry,[8] showing that hsp60 is abundantly expressed in placenta; however, antibodies to hsp60 have not been studied in that work.

In untreated mothers completing their pregnancy, the contamination risk factor for the foetus, which is mildly protected by the maternal antibodies, is 20%–50% for conjunctivitis and 10%–20% for pneumoniae and respiratory disease syndrome[9] with the likely sensitization of the the infants to further chlamydial infections. These findings stress the need for chlamydia detection at 24–26 weeks of gestation.[10]

Pregnancy can be interrupted by chlamydial infection. Most of the studies on current infection[3]

Received September 15, 1996
Accepted October 1, 1996

correlate a late miscarriage with a positive detection. Early spontaneous abortions have been associated with chlamydial antibodies but some authors[11] do not find significant differences with control populations. A recent screening[12] found that chlamydial infection was the second most frequent cause for recurrent foetal losses. Another recent work finds a positive correlation between hsp60 antibodies to the chlamydial 60kD heat shock protein (chsp 60) and spontaneous abortions[14]. More studies are needed to implicate precisely the immunopathogenesis of past or current chlamydial infection in spontaneous abortion.

### ECTOPIC PREGNANCY (EP)

In contrast to infection during pregnancy, numerous authors have associated the risk for ectopic pregnancy with a past chlamydial infection. The mechanism is tubal function impairment or tubal alteration. In fact chlamydial antibody prevalence[13] and chlamydial tubal impairment are the most frequent causes of EP, often revealing an inapparent past infection. Recent studies[15] have demonstrated viable chlamydiae in the tubes by mRNA transcripts. Hsp60 antibodies have been associated with tubal scarring in the pathogenesis of EP[16] through repeated exposures or sensitization to Chlamydia.

After ectopic pregnancy many women undergo IVF because of tubal removal or plasty. But it appears now that chlamydial antigens remain inside the tissues and endometrium possibly affecting IVF outcome.

### IVF

Most of the studies report an unchanged fertilization rate after IVF in chlamydial infected vs. noninfected patients. This can suggest that the oocyte capacity to be fertilized is not impaired by the infection, recent or past even with high antibodies titers. In contrast, after embryo transfer a number of patients either do not reach pregnancy, or suffer early spontaneous abortions. The results are controversial because of the great variability of anti-chlamydia antibody detection methods. Some works have used detection (culture, antigen detection DNA or mRNA detection with or without amplification), others have used chlamydial structural antibodies, or anti hsp60 antibodies, in serum or genital fluids, with various degrees of specificity (genus specific antibodies, genus or bacterial specific hsp). In some studies[17,18,19,20,21,22], there was no correlation between pregnancy rate and the presence of antichlamydia antibodies. In contrast, other authors found a negative correlation between anti chlamydia antibodies and a successful IVF outcome[23,24,25,26,27,28,29]. Because of the established relation between tubal infertility and hsp60 antibodies[30,31,32,33] it seemed possible to hypothesize a link between hsp60 antibodies and a reduced pregnancy rate.

Hsp60, stress response protein for both chlamydia and host[34,35], is a potent immunogenic agent leading to antibody secretion, and inflammation induction[36,37] leading to cytokine secretion through antigen presenting cells.[38] Being homologous to the human hsp60, bacterial hsp60 can trigger autoantibody formation in the host, and development of delayed hypersensitivity reactions building tissue damage in the upper genital tract and also in remote areas like joints and liver. Maternal or embryo hsp expression is not normaly harmful to the maintenance of pregnancy, unless the mother has been sensitized previously to a bacterial hsp. In that case, expression of maternal or embryo hsp60 will reactivate the lymphocytes previously sensitized to hsp60, and this will induce a proinflammatory immune response which might lead to immune rejection. After IVF, embryo implantation in a previously infected endometrium, possibly still hiding altered or impotent elementary bodies, is a challenge.

Embryo and decidua secrete proinflammatory and anti inflammatory cytokines like TNFα, IL, growth factors, and different kinds of hsp among which is hsp60. Recent studies on animal models demonstrate that implantation and embryo development are linked to endogenous and exogenous growth factors; uterine modifications triggered by steroid hormones are settled through local growth factors.[39–41] To date four cytokines are known as regulators of uterine proliferation and differentiation, and embryo implantation: EGF, CSF1, LIF, and IL1. The latter is a proinflammatory, metabolic and immunologic cytokine mediating inflammation[42] and is necessary for endometrium-embryo dialogue. Decidua and endothelial cells produce TNFα, another proinflammatory cytokine. The embryo itself produces antigens, cytokines like IFNγ and tau.[43] TNFα, IL10. IFNγ and TNFα are predominant in placentas of aborting foetuses (Th1 immune response). IFNγ activates NK T-cells, IL10 and IFN tau are immunosuppressive and antiabortive (Th2 immune response). Early inflammatory reaction is necessary for implantation (IL1, LIF) but its deregulation can totally damage implantation[39].

Hypersecretion of TNF$\alpha$, produced by a latent infection in the endometrium, as in chronic chlamydiosis, induces other such cytokine production as IL1, IL8, IL6 and CSF. CSF inhibits LIF in infertile women and could cause embryo rejection by PGE2 production, or at high concentrations, inhibit implantation. IL1 has been demonstrated in Chlamydia infected fallopian tube tissue and could act by anti angiogenic properties, opposite it's physiologic proimplantation (pseudo-inflammatory action).

Embryos secrete hsp in response to stress, or physiologically as protein shedding. Hsp60 and 70 have been demonstrated during the pre-and peri-implantatory phase in the embryo.[44] It is thus evident that in the implantation phase immune modulators are already normally present. Even without inflammation, the role of immune mediators in embryo implantation is not precisely known. Following T-cell activation or suppression, an inflammatory reaction can switch from Th1 to Th2. It has been demonstrated that chlamydial antibodies and chlamydial hsp are related to induction of inflammatory cytokines,[37,45] hsp strongly induces T-cell and macrophage activation and cytokine production, and that the immune response is restricted by certain regions of the MHC.[46] Therefore, we may assume that cytokines secreted by infection-activated T-cells and macrophages send a wrong message to the developing embryo and disrupt the balance between pro and anti-inflammatory cytokines, not only impairing embryo growth, but also suppressing embryo rejection by the maternal tissue. Furthermore, as in other immuno-inflammatory processes, subsequent chronic inflammation, and autoimmune reaction may be due to a non-specific stimulus, of chlamydial, microbial, or other hsp origin[47] cross reacting with not only maternal but also embryo's hsps.

A partner's chlamydial infection can also play a role in the female's rejection of the embryo: chlamydial auto antibody-coated sperm activate, after intercourse, women's T-cells which in turn produce inflammatory cytokines.[48] High levels of inflammatory cytokines like IL6 have been found in men affected with chlamydia-associated prostatitis[49], which also activate the woman's genital tract T-cells during and after intercourse.

According to this hypothesis, to link embryo rejection to chlamydial infection, we had to demonstrate that an inapparent or subclinical inflammation could permanently activate T cells, macrophages and B-cells by presenting altered antigens and heat shock proteins to secrete IFN$\gamma$ and TNF$\alpha$ through interleukin activation. In a recent prospective study, we have attempted to look for evidence of inflammation in the cervical mucus related to local chlamydial IgA, and we found significant correlation. Chlamydial antigenicity has been proven by local specific secretory antibodies, although circulating antibodies were not found. Those patients with a past chlamydial infection had significant titers of inflammatory cytokines. The correlation between inflammatory cytokines, chlamydial hsp, IgA, and a poor pregnancy outcome has been recently demonstrated[50]. The possiblity that a past chlamydial infection enhances Th2 inflammatory processes during early embryo implantation and thus impairs it is then assessed. But this antigen presentation is MHC restricted. Recently[51] it has been shown that genetic susceptibility or resistance to tubal damage in CT infected macaques was correlated to MHC class I antigens. Similarly susceptibility to endometriosis has been associated with expression of MHC class I antigens, which regulate the resistance to lysis by NK T cells. Class II MHC, TNF$\alpha$ and HSP70 genes are very closely located on the same chromosome and play a role in up- and down-regulation of antigenicity, inflammation, and autoimmune-delayed hypersensitivity.[36] MHC class I antigens as well as polymorphism in the gene encoding TNF$\alpha$ has been associated with severe scarring in trachoma, confirming the genotypic susceptibility to severe infection.[52] MHC1 antigen expression also modulates the embryo's susceptibility or resistance to lysis by NK T-cells. If it certainly is necessary to develop research on MHC-related immune reactions towards chronic Chlamydial infection to predict IVF outcome, it seems mandatory to look for inflammation by screening for local chlamydial antibodies, and not only in *Chlamydia trachomatis* seropositive patients. A recent work confirmed the correlation between MHC antigens, antibodies to hsp60 and significant adverse sequelae[53]. Although controversial results on the relation between hsp60 antibodies and pregnancy rates have been reported[54,25] with significant methodological differences between studies, it seems more likely, that hsp60 antibodies are associated with adverse IVF outcome or early spontaneous abortions[25]. In this study, the lack of correlation between pregnancy rate and serologic evidence of past chlamydial infection is consistent with the findings of Claman[54]. Our results, like other works (50), correlate the secretory IgA CT-positivity to inflammation.

To see if chronic chlamydial infection could predict IVF outcome, we studied a series of infertile couples. In an ongoing retrospective study, we looked for antibodies to hsp60 and 70 from chlamydial, *E. coli*, and human origin in those patients. So far, the results are surprising in terms of the relation between past chlamydial infection, infertility, and IVF outcome. We tried to see in what way a past chlamydial infection could impair assisted reproduction attempts in infertile patients undergoing IVF. Some CT seropositive women did not have anti-chsp60 antibodies, some had anti-chsp antibodies and/or anti *E. coli* hsp60 antibodies, and some of them had only anti-human hsp60 antibodies. Those first results already suggest that all past chlamydial infections do not lead to chsp60 antibodies, and that chsp antibodies are associated with chronicity and unsuccessful treatment. Secondly, some infected patients may have only *E. coli* hsp60, but still have human hsp antibodies. A recent study (in press) found that women having human hsp60 antibodies were also reactive with a conserved epitope of chlamydial hsp60, showing that presensitization is sufficient for one or the other hsp60 to launch the antibody reaction, and infected women respond differently according to their genetic susceptibility. This supports the facts already reported that hsp60 antibodies are associated with severe sequelae of chronic upper genital tract chlamydial infections[55,56].

I can conclude that neither anti-structural chlamydia antibodies or anti-chsp60 antibodies are the golden marker to predict IVF outcome in Chlamydia infected couples. We need more prospective studies to define the marker, or the association of markers. After that, other studies will also be necessary to know how to improve the prognosis of IVF outcome by antibiotic, anti-inflammatory or immunomodulating treatments.

### REFERENCES

1. Witkin SS, Bongiovanni AM, Inglis SR: Effect of *Chlamydia* trachomatis on pregnancy outcome: Evaluation of infections detected by DNA amplification, antigen detection and cervival IgA antibodies. Proceedings of the 3rd meeting of the European Society for Chlamydia Research, A Stary edr, 198, 1996.
2. Djukic S, Nedjeljkovic M, Pervulov M, et al.: *Chlamydia trachomatis* infection and pregnancy outcome. Proceedings of the 3rd meeting of the European Society for Chlamydia Research, A. Stary edr, 202, 1996.
3. Mc Gregor J, French J, Parker R et al.: Prevention of premature birth by screening and treatment for common genital tract infections: Results of a prospective controlled evaluation. Am J Obstet Gynecol, 173:157–167, 1995.
4. Ismail M, Pridjian G, Hibbard J, et al.: Significance of positive cervical cultures for *C trachomatis* in patients with preterm premature rupture of membranes. Am J Perinatol, 9:368–370, 1992.
5. Gençay M, Koskiniemi M, Saikku P, et al.: *Chlamydia trachomatis* seropositivity during pregnancy is associated with perinatal complications. Clin Infect Dis 21:2, 424–426, 1995.
6. Ngassa P, Egbe J: Maternal genital *Chlamydia trachomatis* infection and the risk of preterm labor. Int J Gynaecol Obstet, 47:241–246, 1994.
7. Claman P, Toye B, Peeling R, et al.: Serologic evidence of *Chlamydia trachomatis* infection and risk of preterm birth. Can Med Assoc J 153:259–262, 1995.
8. Divers MJ, Bulmer JN, Miller D, et al.: Placental heat shock proteins: No immunohistochemical evidence for a differential stress response in preterm labour. Gynecol Obstet Invest, 4:236–243, 1995.
9. Smith, J, Taylor-Robinson D: Infection due to *Chlamydia trachomatis* in pregnancy and the newborn. Baillieres Clin Obstet Gynaecol 7:237–255, 1993.
10. Bell T, Stamm W, Kuo C, et al.: J: Risk of prenatal transmission of *Chlamydia trachomatis* by mode of delivery. Infect 29:165–169, 1994.
11. Witkin SS, Ledger WJ: Antibodies to *Chlamydia trachomatis* in sera of women with recurrent spontaneous abortions. Am J Obstet Gynecol 167:135–139, 1992.
12. Bustos-Lopez HH, Barron-Vallejo J, Garcia-Malvaez B, et al.: Use of a diagnostic prospective algorithm for patients with recurrent miscarriage. Gynecol Obstet Mex, 63:96–101, 1995.
13. Witkin SS: Immune responses to the 60 kd and 70 kd heat shock proteins (hsp) in women: Relation to fertility, history of infections, contraception and antisperm antibodies (Personnal communication).
14. Coste J, Laumon B, Bremond A, et al.: Sexually transmitted diseases as major causes of ectopic pregnancy: Results of a large case-control study in France. Fertil-Steril, 62:289–295, 1994.
15. Gerard H, Branigan P, Minassian S, et al.: Viability of inapparent *Chlamydia trachomatis* in fallopian tubes of ectopic pregnancies. J Soc Gynecol Invest 3:203–A, 1996.
16. Brunham R, Peeling R, Maclean I, et al.: *Chlamydia trachomatis* associated ectopic pregnancy: serologic and histologic correlates. J Infect Dis 165:1076–1081, 1992.

17. Lessing JB, Kletter Y, Amster R, et al.: Success rates in in vitro fertilization treatment and its correlation with high titer antibodies for *Chlamydia trachomatis.* Isr J Med Sci 27:546–549, 1991.
18. Osser S, Persson K, Wramsby H, et al.: Does previous *Chlamydia trachomatis* infection influence the pregnancy rate of in vitro fertilization and embryo replacement? Am J Obstet Gynecol; 162:40–44, 1990.
19. Coles R: Positive chlamydial serology and its effect on factors influencing outcome of IVF treatment. NZ J Obstet Gynaecol; 31:145–147, 1991.
20. Torode HW, Wheeler PA, Saunders DM, et al.: The role of chlamydial antibodies in an in vitro fertilization program. Fertil Steril 48:87–90, 1987.
21. Asche LV, Kovacs G, Powers JR: Effect of *Chlamydia trachomatis* Infection on Outcome of Treatment of Infertility by IVF. New York: Cambridge University Press, pp. 348–351, 1990.
22. Peek J, Graham F, Hookham A: Prevalence of chlamydial antibodies in women with tubal disease: Impact of *Chlamydia trachomatis* on the demand for in vitro fertilization. N Z Med J 103:63–65, 1990.
23. Rowland G, Forsey T, Steptoe P et al.: Failure of in vitro fertilization and embryo replacement following infection with *Chlamydia trachomatis.* J In Vitro Fert Embryo Transf, 2:151–155, 1985.
24. Agnani G, Goni J, Aksoy S et al.: Influence of Chlamydiae serology and the presence of a pelvic inflammatory state on the results of in vitro fertilization. Rev Fr Gynecol Obstet, 86:327–330, 1991.
25. Witkin SS, Sultan KM, Neal GS, et al.: Unsuspected *Chlamydia trachomatis* infection and in vitro fertilization outcome. Am J Obstet Gynecol 171:1208–1214, 1994.
26. Licciardi F, Grifo JA, Rosenwaks Z, et al.: Relation between antibodies to *Chlamydia trachomatis* and spontaneous abortion following in vitro fertilization. J Assist Reprod Genet 9:207–210, 1992.
27. Rae R, Smith IW, Liston WA, et al.: Chlamydial serologic studies and recurrent spontaneous abortion. Obstet Gynecol 170:783–785, 1994.
28. Lunenfeld E, Shapiro BS, Sarov B, et al.: The association between Chlamydia specific IgG and IgA antibodies and pregnancy outcome in an in vitro fertilization program. J In Vitro Fert Embryo Transf; 6:222–227, 1989.
29. Quinn PA, Petric M, Barkin M, et al.: Prevalence of antibody to *Chlamydia trachomatis* infection in spontaneous abortion and infertility. Am J Obstet Gynecol; 156:291–296, 1987.
30. Toye B, Laferrière C, Claman P, et al.: Association between antibody to the chlamydial heat shock protein and tubal infertility. J Infect Dis; 168:1236–1240, 1993.
31. Arno JN, Yan Y, Clearly RE, et al.: Serologic responses of infertile women to the 60-kd chlamydial heat shock protein (hsp60). Fertil Steril 67:730–735, 1995.
32. Wagar EA, Schachter J, Bavoil P, et al.: Differencial human serologic response to two 60.000 molecular weight *Chlamydia trachomatis* antigens. J Infect Dis 162:922–927, 1990.
33. Brunham RC, Maclean IW, Binns B, et al.: *Chlamydia trachomatis:* Its role in tubal infertility. Infect Dis 152: 1275–1282, 1985.
34. Morrison RP, Belland RJ, Lyng K, et al.: Chlamydial disease pathogenesis. The 54 kD chlamydial hypersensitivity antigen is a stress response protein. J Exp Med 170:1271–1283, 1989.
35. Morrison RP: Immunology of CT infections: immunoprotective and immunopathogenetic mechanisms. In: Quinn TC, ed, Sex trans. dis, NY Raven; 57, 1992.
36. Harloe M, Quayle JA: Heatshock proteins. Friend or foe. Clin Exp Immunol 86:2, 1991.
37. Zhong G, Brunham RC: Antibody responses to the chlamydial heat shock proteins HSP60 and HSP70 are H-2 linked. Infect Immun 60:3143–3149, 1992.
38. Anderton SM, Van der Zee R, Goodacre JA: Inflammation activates self hsp60-specific T cells. Eur J Immunol 23:3–8, 1993.
39. Chaouat G: Immunosuppression précoce et implantation. Contracept Fertil Sex, 23:617–621, 1995.
40. Hazout A: TNF et infection sous-jacente. Contracept Fert Sex, 23:631–634, 1995.
41. Mincheva-Nilsson J, Baranov V, Yeung MM, et al.: Immunomorphologic studies of human decidua-associated lymphoid cells in normal early pregnancy. J Immuno 152:2010–2032, 1994.
42. Stewart CL, Abbondanzo SJ, Cullinan EB: Régulation par les cytokines d'origine maternelle du développement pré-implantatoire et de la réceptivité utérine. Contracept Fertil Sex: 23:555–561, 1995.
43. Martal J, De La Llosa-Hermier MP, Chêne N, et al.: Les Interférons trophoblastiques et la tolérance immunologique embryonnaire. Contracept Fertil Sex, 23:562–572, 1995.
44. Mirkes PE, Grace RH, Little SA: Developmental regulation of heat shock protein synthesis and HSP70 RNA accumulation during postimplantation rat embryogenesis. Teratology 44:77–89, 1991.
45. Witkin SS: Relation between tumor necrosis factor in the endocervix and pregnancy history. (submitted).
46. Askienazy-Elbhar M, Henry-Suchet J: Inflammation post infectieuse dans le syndrome inflammatoire pelvien. Gynecologie Internationale 3:8–10, 1994.
47. Geboes K: From inflammation to lesion. Acta Gastroenterol Belg 57:273–284, 1994.
48. Munoz MG, Witkin SS: Activation of circulating T lymphocytes by autologous sperm from men sensitized to sperm. J Reproduct Immunol, 25:265–275, 1993.
49. Mazzoli S, Meacci F, Salis S, et al.: Anti *Chlamydia trachomatis* specific immune response in sera and secretions of patients affected by prostatitis: in vivo production of IgA and IgG secretory IgA, anti LPS antibodies, IgA 1 and IGA 2 subclasses, interleukins 6, 4 and 10. Proceeding of the 3rd meeting of European Society for Chlamydia Research, p. 160, 1996.
50. Witkin S, Bongiovanni AM, Kligaman I, et al.: IgA antibodies to a Conserved Epitope of the Chlamydia trachomatis 60kd Heat Shock Protein (hsp60) in the women undergoing in vitro fertilization (IVF). (in preparation).
51. Lichtenwalner AB, Patton DL, Cosgrove Sweeney YT, et al.: MHC Class I Alleles Associated with Relative Susceptibility to Pelvic Inflammatory Disease in Chlamydia trachomatis-infected Macaques. Proceedings of the 3rd of European Society for Chlamydia Research, 104, 1996.
52. Ward ME: An update on the immunology of chlamydial infection. Proceeding of the 3rd meeting of European Society for Chlamydia Research, 58, 1996.
53. Peeling RW, Bailey RL, Conway DJ, et al.: Antibody response to the chlamydial heat shock protein 60 (CHSP) is associated with scarring trachoma. Proceedings of the 3rd of European Society for Chlamydia Research, 73, 1996.
54. Claman P, Amimi MN, Peeling RW, et al.: Does serologic evidence of remote Chlamydia trachomatis infection and its heat shock protein (CHSP 60) affect in vitro fertilization-embryo transfer outcome. Fertil Steril 65:146–149, 1996.
55. Beatty WL, Byrne GI, Morrison RP: Repeated and persistent infection with Chlamydia and the development of chronic inflammation and disease. Trends-Microbiol, 2:94–98, 1994.
56. Witkin SS, Jeremias J, Toth M, et al.: Cell Mediated immune response to the recombinant 57-kDa heat-shock protein of Chlamydia trachomatis in women with salpingitis. J Infect Dis 167:1379–1383, 1993.

## References

[PDF_00424] Agnani G., Goni J., Aksoy S., el Kaddissi H., Maillet R., Couetdic G., Roux C., Joanne C. (1991). Influence de la sérologie Chlamydiae et de l'existence d'un status inflammatoire pelvien sur les résultats de la fécondation in vitro.. Rev Fr Gynecol Obstet.

[PDF_00449] Arno J. N., Yuan Y., Cleary R. E., Morrison R. P. (1995). Serologic responses of infertile women to the 60-kd chlamydial heat shock protein (hsp60).. Fertil Steril.

[PDF_00536] Beatty W. L., Byrne G. I., Morrison R. P. (1994). Repeated and persistent infection with Chlamydia and the development of chronic inflammation and disease.. Trends Microbiol.

[PDF_00371] Bell T. A., Stamm W. E., Kuo C. C., Wang S. P., Holmes K. K., Grayston J. T. (1994). Risk of perinatal transmission of Chlamydia trachomatis by mode of delivery.. J Infect.

[PDF_00456] Brunham R. C., Maclean I. W., Binns B., Peeling R. W. (1985). Chlamydia trachomatis: its role in tubal infertility.. J Infect Dis.

[PDF_00393] Brunham R. C., Peeling R., Maclean I., Kosseim M. L., Paraskevas M. (1992). Chlamydia trachomatis-associated ectopic pregnancy: serologic and histologic correlates.. J Infect Dis.

[PDF_00377] Bustos López H. H., Barrón Vallejo J., García Malváez B., Kably Ambe A., Cáceres Zelaya H. (1995). Aplicación de un algoritmo diagnóstico prospectivo para pacientes con pérdida fetal recurrente.. Ginecol Obstet Mex.

[PDF_00474] Chaouat G. (1995). Immunosuppression précoce et implantation.. Contracept Fertil Sex.

[PDF_00531] Claman P., Amimi M. N., Peeling R. W., Toye B., Jessamine P. (1996). Does serologic evidence of remote Chlamydia trachomatis infection and its heat shock protein (CHSP 60) affect in vitro fertilization-embryo transfer outcome?. Fertil Steril.

[PDF_00361] Claman P., Toye B., Peeling R. W., Jessamine P., Belcher J. (1995). Serologic evidence of Chlamydia trachomatis infection and risk of preterm birth.. CMAJ.

[PDF_00385] Coste J., Laumon B., Brémond A., Collet P., Job-Spira N. (1994). Sexually transmitted diseases as major causes of ectopic pregnancy: results from a large case-control study in France.. Fertil Steril.

[PDF_00406] Driscoll G. L., Dodd J., Tyler J. P., Howard B., Lamont B., Coles R. (1991). Positive chlamydial serology and its effect on factors influencing outcome of IVF treatment.. Aust N Z J Obstet Gynaecol.

[PDF_00499] Geboes K. (1994). From inflammation to lesion.. Acta Gastroenterol Belg.

[PDF_00354] Gencay M., Koskiniemi M., Saikku P., Puolakkainen M., Raivio K., Koskela P., Vaheri A. (1995). Chlamydia trachomatis seropositivity during pregnancy is associated with perinatal complications.. Clin Infect Dis.

[PDF_00466] Harboe M., Quayle A. J. (1991). Heat shock proteins: friend and foe?. Clin Exp Immunol.

[PDF_00476] Hazout A. (1995). TNF et infection sous-jacente.. Contracept Fertil Sex.

[PDF_00350] Ismail M. A., Pridjian G., Hibbard J. U., Harth C., Moawad A. A. (1992). Significance of positive cervical cultures for Chlamydia trachomatis in patients with preterm premature rupture of membranes.. Am J Perinatol.

[PDF_00396] Lessing J. B., Kletter Y., Amster R., Amit A., Peyser M. R., Berger S. (1991). Success rates in in vitro fertilization treatment and its correlation with high titer antibodies for Chlamydia trachomatis.. Isr J Med Sci.

[PDF_00431] Licciardi F., Grifo J. A., Rosenwaks Z., Witkin S. S. (1992). Relation between antibodies to Chlamydia trachomatis and spontaneous abortion following in vitro fertilization.. J Assist Reprod Genet.

[PDF_00438] Lunenfeld E., Shapiro B. S., Sarov B., Sarov I., Insler V., Decherney A. H. (1989). The association between chlamydial-specific IgG and IgA antibodies and pregnancy outcome in an in vitro fertilization program.. J In Vitro Fert Embryo Transf.

[PDF_00484] Martal J., De La Llosa-Hermier M. P., Chêne N., Huynh L., Millet S., L'Haridon R., Assal A., Roignant N., Chaouat G. (1995). Les interférons trophoblastiques et la tolérance immunologique embryonnaire.. Contracept Fertil Sex.

[PDF_00345] McGregor J. A., French J. I., Parker R., Draper D., Patterson E., Jones W., Thorsgard K., McFee J. (1995). Prevention of premature birth by screening and treatment for common genital tract infections: results of a prospective controlled evaluation.. Am J Obstet Gynecol.

[PDF_00478] Mincheva-Nilsson L., Baranov V., Yeung M. M., Hammarström S., Hammarström M. L. (1994). Immunomorphologic studies of human decidua-associated lymphoid cells in normal early pregnancy.. J Immunol.

[PDF_00490] Mirkes P. E., Grace R. H., Little S. A. (1991). Developmental regulation of heat shock protein synthesis and HSP 70 RNA accumulation during postimplantation rat embryogenesis.. Teratology.

[PDF_00459] Morrison R. P., Belland R. J., Lyng K., Caldwell H. D. (1989). Chlamydial disease pathogenesis. The 57-kD chlamydial hypersensitivity antigen is a stress response protein.. J Exp Med.

[PDF_00501] Munoz M. G., Witkin S. S. (1993). Activation of circulating gamma delta T-lymphocytes by autologous sperm from men sensitized to sperm.. J Reprod Immunol.

[PDF_00358] Ngassa P. C., Egbe J. A. (1994). Maternal genital Chlamydia trachomatis infection and the risk of preterm labor.. Int J Gynaecol Obstet.

[PDF_00402] Osser S., Persson K., Wramsby H., Liedholm P. (1990). Dose previous Chlamydia trachomatis infection influence the pregnancy rate of in vitro fertilization and embryo replacement?. Am J Obstet Gynecol.

[PDF_00416] Peek J. C., Graham F. M., Hookham A. (1990). Prevalence of chlamydial antibodies in women with tubal disease: impact of Chlamydia trachomatis on the demand for in vitro fertilisation.. N Z Med J.

[PDF_00442] Quinn P. A., Petric M., Barkin M., Butany J., Derzko C., Gysler M., Lie K. I., Shewchuck A. B., Shuber J., Ryan E. (1987). Prevalence of antibody to Chlamydia trachomatis in spontaneous abortion and infertility.. Am J Obstet Gynecol.

[PDF_00435] Rae R., Smith I. W., Liston W. A., Kilpatrick D. C. (1994). Chlamydial serologic studies and recurrent spontaneous abortion.. Am J Obstet Gynecol.

[PDF_00420] Rowland G. F., Forsey T., Moss T. R., Steptoe P. C., Hewitt J., Darougar S. (1985). Failure of in vitro fertilization and embryo replacement following infection with Chlamydia trachomatis.. J In Vitro Fert Embryo Transf.

[PDF_00368] Smith J. R., Taylor-Robinson D. (1993). Infection due to Chlamydia trachomatis in pregnancy and the newborn.. Baillieres Clin Obstet Gynaecol.

[PDF_00482] Stewart C. L., Abbondanzo S. J., Cullinan E. B. (1995). Régulation par les cytokines d'origine maternelle du développement pré-implantatoire et de la réceptivité utérine.. Contracept Fertil Sex.

[PDF_00446] Toye B., Laferrière C., Claman P., Jessamine P., Peeling R. (1993). Association between antibody to the chlamydial heat-shock protein and tubal infertility.. J Infect Dis.

[PDF_00452] Wagar E. A., Schachter J., Bavoil P., Stephens R. S. (1990). Differential human serologic response to two 60,000 molecular weight Chlamydia trachomatis antigens.. J Infect Dis.

[PDF_00540] Witkin S. S., Jeremias J., Toth M., Ledger W. J. (1993). Cell-mediated immune response to the recombinant 57-kDa heat-shock protein of Chlamydia trachomatis in women with salpingitis.. J Infect Dis.

[PDF_00374] Witkin S. S., Ledger W. J. (1992). Antibodies to Chlamydia trachomatis in sera of women with recurrent spontaneous abortions.. Am J Obstet Gynecol.

[PDF_00428] Witkin S. S., Sultan K. M., Neal G. S., Jeremias J., Grifo J. A., Rosenwaks Z. (1994). Unsuspected Chlamydia trachomatis infection and in vitro fertilization outcome.. Am J Obstet Gynecol.

[PDF_00468] Zhong G., Brunham R. C. (1992). Antibody responses to the chlamydial heat shock proteins hsp60 and hsp70 are H-2 linked.. Infect Immun.

